# Association between non-highdensity lipoprotein cholesterol to high-density lipoprotein cholesterol ratio and cardiovascular-kidney-metabolic syndrome: evidence from NHANES 2001–2018

**DOI:** 10.3389/fnut.2025.1548851

**Published:** 2025-02-28

**Authors:** Yang Duan, Ke Yang, Tianai Zhang, Xiangsheng Guo, Qianran Yin, He Liu

**Affiliations:** ^1^Department of Cardiology, The Affiliated Hospital of Xuzhou Medical University, Xuzhou, China; ^2^Department of Cardiology, The Fourth Affiliated Hospital of Soochow University, Suzhou, China; ^3^Guzhen County Traditional Chinese Medicine Hospital, Bengbu, China; ^4^School of Integrative Medicine, Nanjing University of Chinese Medicine, Nanjing, China; ^5^Department of Cardiology, Xuzhou Central Hospital, Xuzhou, China

**Keywords:** NHHR, CKM, RCS, intervention strategies, NHANES

## Abstract

**Objective:**

This research is to analyze the connection between NHHR and CKD occurrence using NHANES from 2001 to 2018. It will evaluate the feasibility of NHHR as a tool for predicting CKM syndrome and offer valuable insights for personalized treatment approaches within the U.S. population.

**Methods:**

Data from 16,575 individuals aged 20 to 69 years were analyzed, having excluded those who were pregnant and individuals with incomplete data. CKM syndrome was characterized by the simultaneous presence of CKD and Cardiometabolic Syndrome (CMS). For the statistical analysis, weighted logistic regression models were applied, accounting for variables such as age, gender, ethnicity, educational background, marital status, lifestyle factors, and preexisting health conditions. Differently, restricted cubic splines (RCS) were applied to investigate any possible nonlinear relationships between NHHR and CKM in the study.

**Results:**

The research revealed that the occurrence of CKM syndrome was more prevalent among individuals aged 60 and older, with women representing 55.36% of those affected. Additionally, NHHR levels were notably elevated in CKM patients when compared to those without CKM (*p* < 0.0001). As NHHR increased, the prevalence of CKM also rose, with the highest prevalence in the highest NHHR quartile (Q4: 36.06%). A positive connection between NHHR and CKM was indicated by multivariable logistic regression, especially in the upper quartiles of NHHR (Q3 and Q4). Moreover, RCS analysis displayed a noteworthy nonlinear connection between NHHR and CKM occurrence. The subgroup analysis uncovered significant interactions influenced by BMI and Hypertension.

**Conclusion:**

With the rising global prevalence of CKM syndrome, early identification of high-risk individuals using NHHR could inform targeted prevention and intervention strategies. Future research should focus on validating NHHR in diverse populations and exploring its clinical utility, as well as examining its relationship with other biomarkers of metabolic dysfunction to better understand CKM syndrome’s complex pathophysiology.

## Introduction

In recent years, the cardiovascular-kidney-metabolic (CKM) syndrome has gained attention as a major public health issue worldwide, owing to its profound effect on both disease burden and mortality rates. CKM syndrome refers to the complex interplay of cardiovascular diseases (CVD), chronic kidney disease (CKD), and metabolic conditions like obesity and Diabetes Mellitus (DM) (1, 2). These conditions often coexist, forming a complex web of pathophysiological mechanisms that significantly increase the risks of both morbidity and mortality, particularly through cardiovascular complications. Extensive research has also confirmed the intricate interrelationships between the cardiovascular, renal, and metabolic systems (3). CKD is widely acknowledged as a significant contributor to CVD, with a reduced estimated glomerular filtration rate (eGFR) closely linked to a heightened risk of CVD and mortality (4). Additionally, the incidence of DM among patients with heart failure is approximately 20%, which is roughly four times higher than in those without heart failure, where the prevalence ranges from 4 to 6% (5). Recent data also shows that nearly 40% of individuals with DM are concurrently affected by CKD (6). In recent decades, the prevalence of CKM syndrome has risen dramatically, driven largely by demographic changes such as aging populations and an increase in lifestyle-related risk factors, including sedentary behavior, unhealthy diets, and rising obesity rates (7). As a result, CKM syndrome has become a major public health challenge worldwide, requiring more comprehensive strategies for early identification, prevention, and management of its associated risks.

The pathogenesis of CKM syndrome is complex, involving metabolic disturbances, chronic low-grade inflammation, endothelial dysfunction, and other contributing factors. Dyslipidemia is considered a significant to the onset of CKM syndrome, with the low-density lipoprotein cholesterol (LDL-C) to high-density lipoprotein cholesterol (HDL-C) ratio being particularly influential. This ratio, as a key marker of lipid metabolism, can well identify and predict the CKM syndrome (8). Recently, the non-high-density lipoprotein cholesterol to high-density lipoprotein cholesterol ratio (NHHR) has garnered significant attention as a novel biomarker for evaluating health risks. NHHR serves as an indicator of an individual’s overall lipid metabolic health and is strongly linked to several chronic conditions. Studies demonstrate that higher NHHR would cause an increased risk of CVD, metabolic syndrome (MetS), and CKD (9–12). With the global increase in obesity and DM, NHHR has become an important focus of academic research as a potential indicator of metabolic disease risk.

While research has demonstrated the connection between NHHR and other severe diseases, the precise correlation with CKM syndrome remains inadequately examined. Consequently, this study intends to examine NHHR and the occurrence of CKD utilizing data sourced from the National Health and Nutrition Examination Survey (NHANES) spanning the years 2001–2018. The ultimate goal is to assess NHHR as a predictive indicator to guide individualized treatment strategies for CKM syndrome. In addition, we will consider various factors, such as age, gender, body mass index (BMI), and blood pressure, to provide more reliable evidence for the early identification and intervention of CKM syndrome.

## Materials and methods

### Research design and subjects

The NHANES, conducted by the National Center for Health Statistics (NCHS) at the Centers for Disease Control and Prevention (CDC), a cross-sectional, multi-stage probability survey designed to assess the health and nutrition status of individuals in the United States (13). The study protocol was approved by the NCHS Ethics Review Board, and all participants provided written informed consent. This analysis is based on data from the NHANES dataset covering the period from 2001 to 2018, which is available (14).^1^

**Figure 1:**
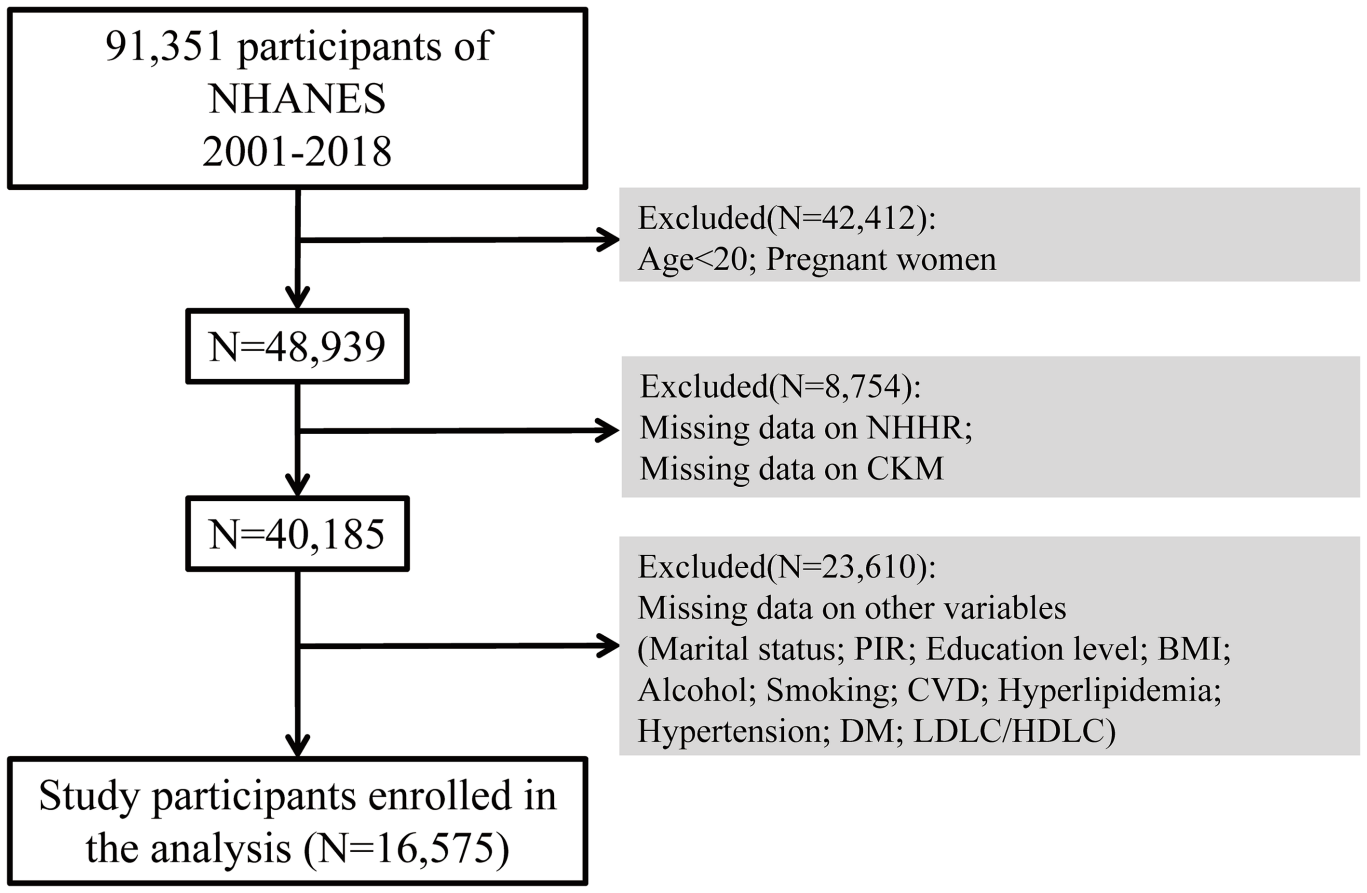
Flowchart displaying the selection of participants.

Initially, there were 91,351 participants who took part in the study. Throughout the selection process, 42,412 individuals were removed because they were either under 20 years old or pregnant. Additionally, 8,754 participants were excluded due to absent NHHR or CKM data, while 23,610 were excluded due to missing data on covariates including marital status, income-to-poverty ratio, education level, body mass index (BMI), alcohol consumption, smoking habits, CVD, hyperlipidemia, hypertension, and diabetes mellitus (DM), LDL-C/HDL-C. As a result, the analysis ultimately included 16,575 qualified participants. The sample selection method is illustrated in Figure 1.

### Diagnosis approach to cardiovascular-kidney-metabolic syndrome

CKM syndrome is presently without a standardized diagnostic criterion. In this research, CKM syndrome is characterized by the simultaneous presence of CKD and Cardiometabolic Syndrome (CMS) (15). The diagnosis of Metabolic Syndrome (MS) was based on the NCEP-ATP III criteria, which require the presence of at least three of the following five factors: central obesity (waist circumference ≥ 102 cm for men or ≥ 88 cm for women), elevated triglycerides (≥150 mg/dL), low HDL cholesterol (HDL-C < 40 mg/dL for men or < 50 mg/dL for women), hypertension (systolic BP ≥140 mmHg or diastolic BP ≥90 mmHg), and hyperglycemia (fasting plasma glucose ≥100 mg/dL or use of antidiabetic medications). CKD was identified when the estimated Glomerular Filtration Rate (eGFR) fell below 60 mL/min/1.73 m^2^, or when the urinary albumin-to-creatinine ratio (UACR) exceeded 30 mg/g. The eGFR was determined utilizing the CKD-EPI creatinine equation established in 2009 (16).

### Demographic characteristics and other covariates

The study considered a range of covariates, including factors such as race and ethnicity, educational level, marital status, the poverty-income ratio (PIR), smoking and alcohol consumption habits, physical activity levels, and various metabolic and cardiovascular health conditions. Ethnicity or race was classified as Mexican American, Non-Hispanic White, Non-Hispanic Black, and other racial categories. Educational attainment was classified into three categories: less than high school, high school graduate, and higher than high school. Marital status was divided into groups such as married or living with a partner, widowed/divorced/separated, and never married. The Poverty Income Ratio (PIR) reflected family income relative to the federal poverty line, adjusting for inflation and family size.

Smoking habits were categorized into three groups: individuals who have never smoked (defined as consuming fewer than 100 cigarettes in their lifetime), those who are former smokers (those who have smoked more than 100 cigarettes but no longer do), and current smokers (individuals who have smoked more than 100 cigarettes and continue to smoke). Alcohol consumption was classified into five groups: non-drinkers, former drinkers, light drinkers (females ≤1 drink per occasion, males ≤2 drinks per occasion), moderate drinkers (females 2–3 drinks per occasion, males 3–4 drinks per occasion, or individuals who binge drink at least twice a month), and heavy drinkers (females ≥4 drinks/session, males ≥5 drinks/session, or at least 5 binge drinking episodes per month) (17). For blood pressure measurement, the average SBP and DBP were calculated from multiple readings, excluding the first measurement. Diastolic values of zero were not used in the calculation; If every diastolic measurement registered as zero, the mean would be deemed as zero, particularly when there is just a single blood pressure reading, it was considered as the average. Individuals diagnosed with hypertension by a healthcare provider or were undergoing antihypertensive therapy were also included in the hypertension group. Waist circumference, weight, and height were measured following established protocols, while serum TG and HDL-C levels were determined, and fasting plasma glucose was measured in plasma. Hyperlipidemia was defined by any of the following criteria: triglyceride levels ≥150 mg/dL, total cholesterol ≥200 mg/dL, LDL cholesterol ≥130 mg/dL, or HDL cholesterol levels (men <40 mg/dL, women <50 mg/dL), or the use of medications aimed at lowering lipid levels. DM was diagnosed based on one or more of these conditions: a clinical diagnosis of DM, HbA1c ≥6.5%, fasting blood glucose ≥7.0 mmol/L, random blood glucose ≥11.1 mmol/L, a two-hour OGTT blood glucose ≥11.1 mmol/L, or the use of antidiabetic medications or insulin. CVD was characterized by the existence of any of the subsequent conditions: coronary artery disease, congestive heart failure, myocardial infarction, stroke, or angina.

### Statistical analysis

For analyzing categorical data, Chi-square tests were conducted, while Wilcoxon test were applied to assess continuous variables in order to identify differences across groups. To evaluate the relationship between NHHR and CKM syndrome, weighted logistic regression models were utilized. The models incorporated various adjustment variables, including age, sex, race, marital status, PIR, education level, BMI, alcohol use, smoking habits, cardiovascular disease, hypertension, hyperlipidemia, and diabetes mellitus. Additionally, restricted cubic splines (RCS) were used to assess potential nonlinear relationships between NHHR and CKM. The performance of the model is evaluated using the ROC curve, specifically through the AUC value. Additionally, the relationship between NHHR and CKM risk is illustrated via a Nomogram, which enhances intuitive risk prediction.

The data for this study were sourced from the publicly available NHANES datasets, and the process of data collection complied with established ethical standards, which included obtaining written informed consent from every participant. This investigation conformed to pertinent ethical guidelines and operational protocols. All statistical analyses were performed using R software (version 4.3.2) along with the “nhanesR” package (R Core Team, 2023). Statistical significance is defined as a *p* value of less than 0.05.

## Results

### Demographic and clinical characteristics of the participants at baseline

The baseline characteristics of participants with and without CKM from the NHANES dataset between the years 2001 and 2018 are displayed in Table 1. The results indicate a higher prevalence of CKM in individuals aged 60 and above, with females accounting for 55.36% of the patients (*p* < 0.05). Notably, the NHHR of CKM patients was significantly higher than that of non-CKM patients (*p* < 0.0001). Notable variations in other sample characteristics, such as marital status, education, BMI, alcohol use, smoking habits, presence of CVD, hypertension, hyperlipidemia, DM, and LDL-C/HDL-C were also found between the two groups (*p* < 0.05). The range of NHHR quartiles was 0.284–1.912, 1.912–2.622, 2.622–3.514, and 3.514–26.667. The prevalence rates increased from 17.77% in Q1 to 36.06% in Q4. The prevalence of CKM syndrome significantly increased with higher NHHR quartiles.

**Table 1:** Baseline characteristics of participants by CKM from the NHANES, 2001–2018.

Character	Total	No_CKM	CKM	*p* value
Age				< 0.0001
<60	10,865(74.61)	10,401(78.08)	464(33.16)	
≥60	5,710(25.39)	4,418(21.92)	1,292(66.84)	
Sex				< 0.001
Female	8,256(50.54)	7,325(50.14)	931(55.36)	
Male	8,319(49.46)	7,494(49.86)	825(44.64)	
Race				0.07
Mexican American	2,703(7.81)	2,421(7.86)	282(7.23)	
Non-Hispanic White	7,842(71.05)	6,934(70.95)	908(72.24)	
Non-Hispanic Black	3,249(9.99)	2,908(9.90)	341(11.01)	
Others	2,781(11.16)	2,556(11.29)	225(9.51)	
Marital_status				< 0.0001
Married or living with a partner	10,096(64.88)	9,097(65.12)	999(62.02)	
Widowed/divorced/separated	3,648(18.09)	3,011(16.94)	637(31.81)	
Never married	2,831(17.03)	2,711(17.94)	120(6.17)	
Education_level				< 0.0001
Less than High school	4,046(15.65)	3,450(14.96)	596(23.90)	
High school	3,843(23.79)	3,385(23.28)	458(30.00)	
More than High school	8,686(60.56)	7,984(61.76)	702(46.11)	
PIR				0.08
<1	3,189(12.96)	2,817(12.81)	372(14.74)	
≥1	13,386(87.04)	12,002(87.19)	1,384(85.26)	
BMI				< 0.0001
<25	4,969(31.14)	4,791(33.03)	178(8.55)	
≥25 to <30	5,614(33.57)	5,051(33.75)	563(31.41)	
≥30	5,992(35.29)	4,977(33.22)	1,015(60.04)	
Alcohol				< 0.0001
Former	2,871(14.24)	2,375(13.30)	496(25.53)	
Never	2,249(10.75)	1933(10.26)	316(16.59)	
Mild	5,716(37.15)	5,121(37.19)	595(36.65)	
Moderate	2,459(17.13)	2,317(17.77)	142(9.45)	
Heavy	3,280(20.72)	3,073(21.47)	207(11.78)	
Smoking				< 0.0001
Former	4,250(25.71)	3,619(24.85)	631(35.97)	
Never	8,870(53.37)	8,013(53.81)	857(48.10)	
Now	3,455(20.92)	3,187(21.34)	268(15.93)	
CVD				< 0.0001
No	14,728(91.27)	13,544(93.10)	1,184(69.46)	
Yes	1847(8.73)	1,275(6.90)	572(30.54)	
Hyperlipidemia				< 0.0001
No	4,578(28.91)	4,454(30.70)	124(7.44)	
Yes	11,997(71.09)	10,365(69.30)	1,632(92.56)	
Hypertension				< 0.0001
No	9,500(62.29)	9,202(66.09)	298(16.84)	
Yes	7,075(37.71)	5,617(33.91)	1,458(83.16)	
DM				< 0.0001
No	13,402(85.63)	12,631(88.77)	771(48.15)	
Yes	3,173(14.37)	2,188(11.23)	985(51.85)	
NHHR	2.62(1.91,3.51)	2.58(1.89,3.47)	3.05(2.18,3.93)	< 0.0001
NHHR_Q				< 0.0001
Q1 [0.284,1.912]	4,141(25.17)	3,839(25.79)	302(17.77)	
Q2 [1.912,2.622]	4,143(25.02)	3,795(25.53)	348(19.01)	
Q3 [2.622,3.514]	4,148(24.87)	3,665(24.68)	483(27.16)	
Q4 [3.514,26.667]	4,143(24.94)	3,520(24.01)	623(36.06)	
LDL-C/HDL-C	2.15(1.59,2.85)	2.14(1.59,2.83)	2.33(1.65,3.02)	< 0.0001

Median (IQR) for continuous; n (%) for categorical (Percent values were weighted to account for the complex survey design). Wilcoxon test for continuous; chi-squared test for categorical.

### The relationship between NHHR and CKM

Table 2 illustrates the relationship between NHHR and CKM, evaluated through multivariable logistic regression models, both with and without the inclusion of covariates. In this evaluation, model 1 adjusted for demographic factors (age, gender, and race); model 2 additionally incorporated more demographic and socioeconomic variables (marital status, education level, PIR, BMI, alcohol, and smoking); while model 3 included all potential confounders outlined in the methods section. When using Q1 as the reference group, model 1 identifies a notable positive relationship between NHHR and CKM in the Q2, Q3, and Q4 categories (Model 1: OR, 1.463; 95% CI: 1.383, 1.547; *p* < 0.05). Similarly, both models 2 and 3 continue to show a strong positive link between NHHR and CKM in the Q4 categories (Model 2: OR, 1.298; 95% CI: 1.216, 1.386; *p* < 0.05; Model 3: OR, 1.270; 95% CI: 1.179, 1.369; *p* < 0.05). When using Q1 as the control group, a significant positive correlation persists between NHHR Q4 (3.514, 26.667) and CKM in Model 3, with an odds ratio (OR) of 1.836; 95% confidence interval (CI): 1.418, 2.377; *p* < 0.05. Additionally, the trend test yields a value less than 0.05.

**Table 2:** Relationship between NHHR and CKM in the logistic regression models from the NHANES, 2001–2018.

Character	Crude model		Model 1		Model 2		Model 3	
	OR (95%CI)	*p* value	OR (95%CI)	*p* value	OR (95%CI)	*p* value	OR (95%CI)	*p* value
NHHR	1.275(1.215,1.339)	<0.0001	1.463(1.383,1.547)	<0.0001	1.298(1.216,1.386)	<0.0001	1.270(1.179,1.369)	<0.0001
NHHR (Quartile)								
Q1 [0.284,1.912]	ref		ref		ref		ref	
Q2 [1.912,2.622]	1.080(0.866,1.348)	0.490	1.136(0.897,1.439)	0.286	0.876(0.676,1.134)	0.311	0.867(0.660,1.139)	0.303
Q3 [2.622,3.514]	1.597(1.250,2.039)	<0.001	1.939(1.492,2.521)	<0.0001	1.288(0.971,1.708)	0.078	1.237(0.932,1.641)	0.139
Q4 [3.514,26.667]	2.179(1.818,2.610)	<0.0001	3.260(2.661,3.995)	<0.0001	1.994(1.584,2.508)	<0.0001	1.836(1.418,2.377)	<0.0001
*P* for trend		<0.0001		<0.0001		<0.0001		<0.0001

Model 1: Age, Sex, Race. Model 2: Age, Sex, Race, Marital_status, PIR, Education_level, BMI, Alcohol, Smoking. Model 3: Age, Sex, Race, Marital_status, PIR, Education_level, BMI, Alcohol, Smoking, CVD, Hypertension, Hyperlipidemia, DM.

### Nonlinear association between NHHR and CKM

**Figure 2:**
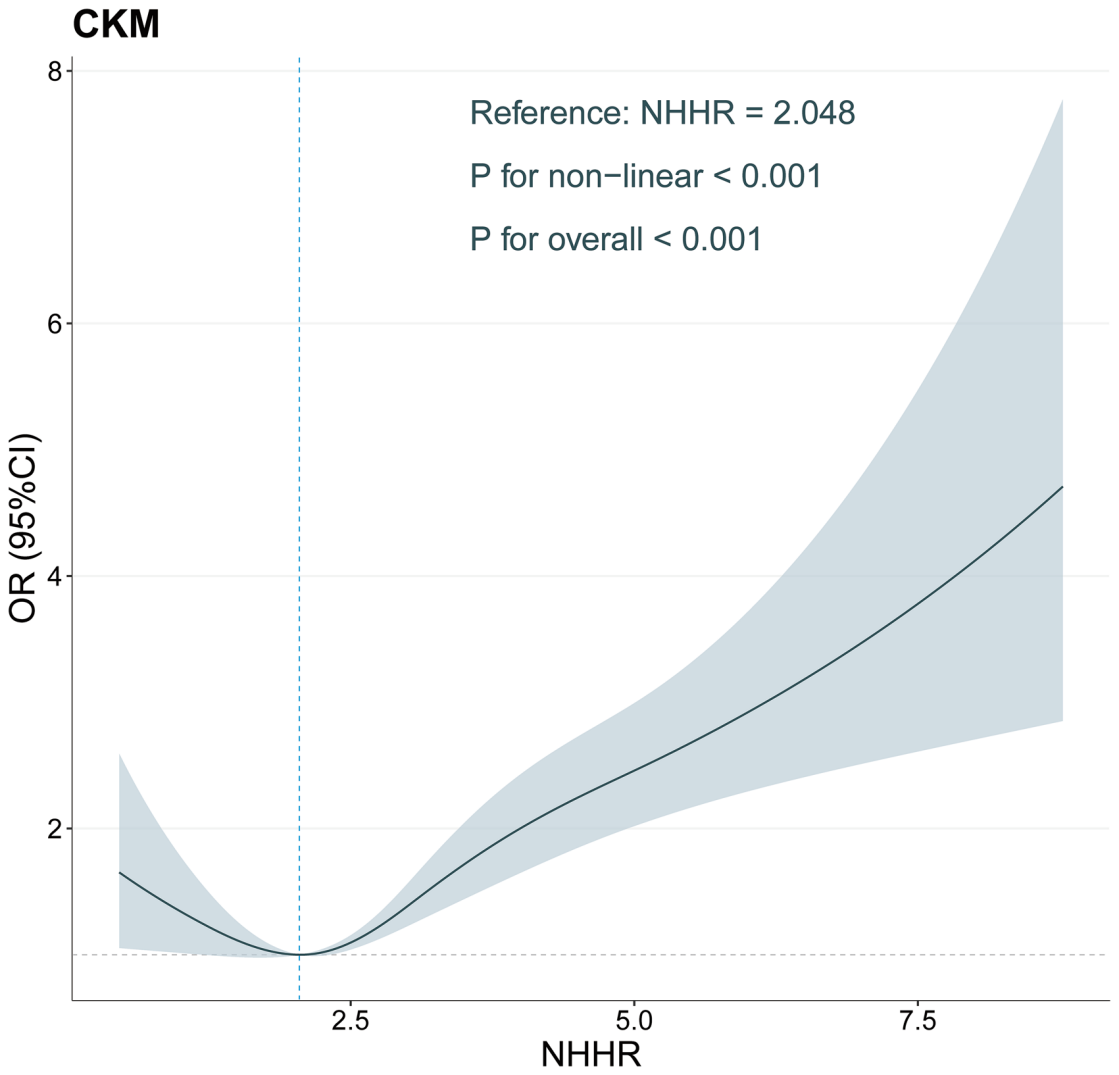
Restricted cubic spline of the association between NHHR levels and CKM adjusted for all covariates.

In the analysis, we used RCS regression analysis to explore the nonlinear relationship between NHHR and CKM (Figure 2). The findings indicated a notable nonlinear correlation between NHHR and CKM (*p* < 0.001), and the overall relationship was highly statistically significant (*p* < 0.001). Specifically, as NHHR increased, the OR for CKM risk gradually increased, with a marked rise in CKM risk at higher NHHR levels. However, within the lower range of NHHR, the OR showed a downward trend, suggesting that CKM risk may decrease at lower NHHR values.

### Subgroup analysis

**Figure 3:**
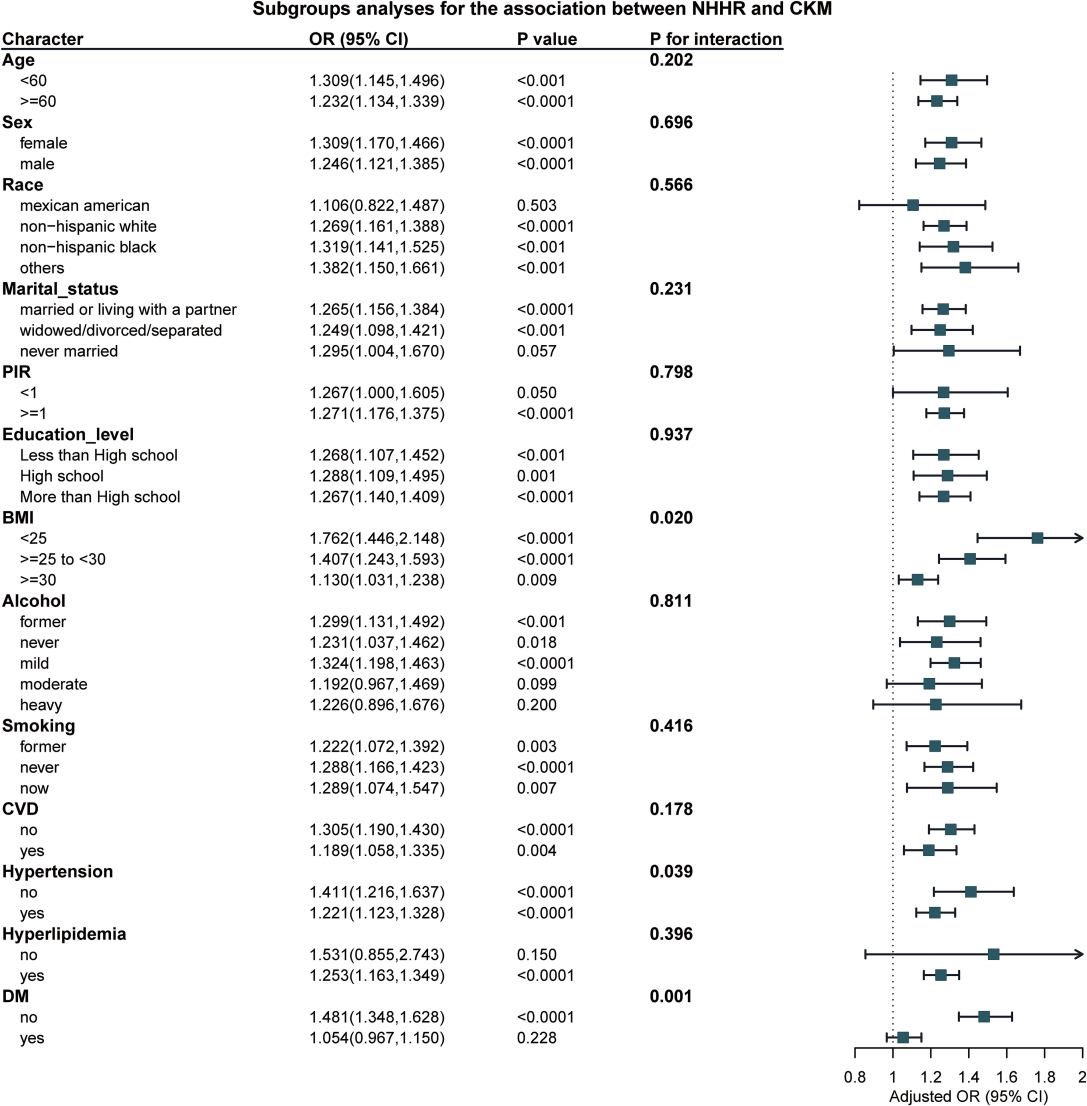
Results of subgroup analysis.

In the subgroup analysis, our results show a significant interaction between BMI and Hypertension (with P for interaction = 0.02 for BMI and P for interaction = 0.007 for Hypertension, as shown in Table 3 and Figure 3). This indicates that with the increase in NHHR, the risk of CKM also rises. However, no such difference was observed in other subgroups.

**Table 3:** Results of subgroup analysis.

Character	OR (95% CI)	*p* value	*P* for interaction
Age			0.202
<60	1.309(1.145,1.496)	<0.001	
≥60	1.232(1.134,1.339)	<0.0001	
Sex			0.696
Female	1.309(1.170,1.466)	<0.0001	
Male	1.246(1.121,1.385)	<0.0001	
Race			0.566
Mexican American	1.106(0.822,1.487)	0.503	
Non-Hispanic White	1.269(1.161,1.388)	<0.0001	
Non-Hispanic Black	1.319(1.141,1.525)	<0.001	
Others	1.382(1.150,1.661)	<0.001	
Marital_status			0.231
Married or living with a partner	1.265(1.156,1.384)	<0.0001	
Widowed/divorced/separated	1.249(1.098,1.421)	<0.001	
Never married	1.295(1.004, 1.670)	0.057	
PIR			0.798
<1	1.267(1.000,1.605)	0.050	
≥1	1.271(1.176,1.375)	<0.0001	
Education_level			0.937
Less than High school	1.268(1.107,1.452)	<0.001	
High school	1.288(1.109,1.495)	0.001	
More than High school	1.267(1.140,1.409)	<0.0001	
BMI			0.020
<25	1.762(1.446,2.148)	<0.0001	
≥25 to <30	1.407(1.243,1.593)	<0.0001	
≥30	1.130(1.031,1.238)	0.009	
Alcohol			0.811
Former	1.299(1.131,1.492)	<0.001	
Never	1.231(1.037,1.462)	0.018	
Mild	1.324(1.198,1.463)	<0.0001	
Moderate	1.192(0.967,1.469)	0.099	
Heavy	1.226(0.896, 1.676)	0.200	
Smoking			0.416
Former	1.222(1.072,1.392)	0.003	
Never	1.288(1.166,1.423)	<0.0001	
Now	1.289(1.074,1.547)	0.007	
CVD			0.178
No	1.305(1.190,1.430)	<0.0001	
Yes	1.189(1.058,1.335)	0.004	
Hypertension			0.039
No	1.411(1.216,1.637)	<0.0001	
Yes	1.221(1.123,1.328)	<0.0001	
Hyperlipidemia			0.396
No	1.531(0.855,2.743)	0.150	
Yes	1.253(1.163,1.349)	<0.0001	
DM			0.001
No	1.481(1.348,1.628)	<0.0001	
Yes	1.054(0.967,1.150)	0.228	

### ROC curve analysis and nomogram model

**Figure 4:**
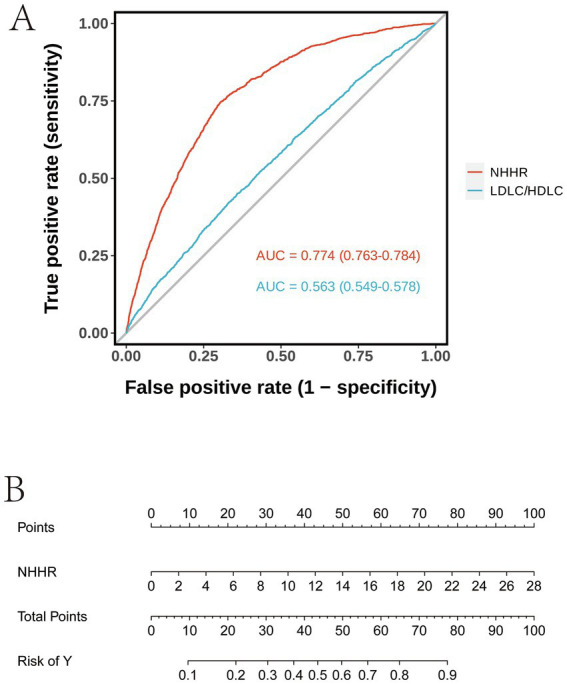
**(A)** ROC curve analysis **(B)** Nomogram model analysis.

The ROC curve in Figure 4A demonstrates that the AUC values for the two models are 0.774 (95% CI: 0.763–0.784) and 0.563 (95% CI: 0.549–0.578), respectively. A higher AUC value indicates superior classification performance of the model. The NHHR model exhibits a higher AUC value, suggesting that its classification performance surpasses that of the LDL-C/HDL-C model. The Nomogram in Figure 4B illustrates the point distribution for NHHR and the corresponding relationship between total points and risk. This Nomogram allows for an intuitive visualization of the contribution of various variable values to the total points, as well as the linear relationship between total points and risk.

## Discussion

The growing global burden of CKM syndrome has prompted significant interest in identifying reliable biomarkers that can predict its onset and progression. This research employed information from the NHANES program, which took place from 2001 to 2018, representing a distinctive dataset that offers in-depth perspectives on the health and nutritional conditions of the U.S. populace. Data was gathered through interviews, standardized physical assessments, and biological samples. As far as we are aware, this is the first in-depth study to explore the relationship between NHHR and CKM syndrome in a large and diverse population. Our findings reveal that, even after accounting for various demographic, socioeconomic, and clinical variables, elevated NHHR values are notably correlated with a greater likelihood of developing CKM syndrome. This association suggests that NHHR, as a comprehensive lipid metabolism biomarker assessing CVD risk, may be a vital factor in the development and progression of CKM syndrome. Analyses of sensitivity provided additional backing for the reliability of this association, enhancing the likelihood of NHHR being an important predictive measure for the risk of CKM syndrome. Our findings align with existing literature, underscoring the importance of systemic health factors in maintaining cardiovascular, renal, and metabolic function. Previous studies have shown that in CKM syndrome mouse models, a Western diet rich in fats, carbohydrates, and salt led to more profound and significant metabolic, cardiac, vascular, and renal dysfunction and damage (8).

The association between NHHR and CKM syndrome can be explained through multiple interconnected pathophysiological mechanisms. Lipid dysregulation, characterized by elevated levels of atherogenic lipoproteins (such as non-HDL-C) and reduced levels of protective HDL-C, contributes to endothelial dysfunction, inflammation, and metabolic disturbances—key features that drive the development of CKM syndrome (18). The latest research suggests that HDL-lncRNA LEXIS may be useful to better identify FH subjects with more pronounced vascular damage (19). Moreover, studies suggest that non-HDL cholesterol serves as a more dependable indicator of cardiovascular incidents compared to conventional lipid indicators, like total cholesterol or LDL cholesterol, emphasizing its significance as a risk element in relation to CKM syndrome. Studies have also shown that, in addition to prominent glucose metabolism abnormalities, diabetic patients often exhibit atherosclerotic lipid abnormalities, characterized by elevated non-HDL-C and triglyceride (TG) levels, along with reduced HDL-C concentrations (12, 20, 21). The advantages of NHHR in cardiovascular disease assessment have also been highlighted (22), recent studies also indicate that NHHR is crucial for independently evaluating the risks associated with non-alcoholic fatty liver disease (NAFLD), CKD, and MetS (23–25).

The findings of our research have substantial relevance for assessing risk, preventing, and treating CKM syndrome. Elevated NHHR typically reflects dyslipidemia and lipid metabolism abnormalities, which may indicate underlying pathological processes such as systemic inflammation, endothelial dysfunction, and atherosclerosis. This insight helps identify individuals at high risk for CKM syndrome at an early stage. By monitoring NHHR, clinicians can develop personalized intervention strategies. For instance, for patients with higher NHHR, interventions like improving lipid metabolism, controlling body weight, and enhancing anti-inflammatory treatments can be employed to reduce the risk of developing CKM syndrome. NHHR not only plays a crucial role in clinical risk assessment but can also serve as a biomarker in clinical trials to evaluate treatment effects. For example, in the development of new lipid-regulating drugs, NHHR could be an important indicator for assessing the drug’s impact, particularly in the treatment of cardiovascular and renal diseases. In conclusion, using NHHR as a predictor of CKM syndrome can enhance the sensitivity of clinical diagnoses and enable early detection, providing a theoretical basis and practical guidance for individualized treatment, preventive management, and drug development.

Our study data indicate that individuals aged over 60 years of age, females, those with lower educational attainment, BMI > 30, a history of CVD, smoking, hyperlipidemia, hypertension, and DM exhibit an elevated NHHR, which in turn impacts the prevalence of CKM syndrome. This paper discusses the elderly population as a case example, highlighting that with age, various physiological systems, particularly the cardiovascular, renal, and metabolic systems, undergo gradual deterioration. Older adults often present with chronic low-grade inflammation, endothelial dysfunction, and metabolic disturbances, all of which collectively contribute to the onset of CKM syndrome.

Specifically, the insulin sensitivity of older adults generally decreases, fat distribution changes, and cardiovascular risk increases. These physiological alterations make older individuals more susceptible to CVD, CKD, and metabolic disorders. The prevalence of CVD rises significantly with age. A cohort study conducted in the Netherlands showed that the incidence of CVD increased with age, from less than 9% in those under 65 years to 28% in those aged ≥65 years (26). Additionally, the occurrence of MetS is markedly elevated in older adults. A study conducted in China involving middle-aged and elderly individuals found that around 42.81% of those aged 65 years and above are diagnosed with MetS (27). In a similar trend, the global incidence of chronic kidney disease among older adults has also increased. A thorough examination of the worldwide impact of chronic kidney disease conducted in 2010 revealed that the rates of CKD stages escalate with advancing age, particularly showing significant growth within the demographic of individuals aged 65 and above. In this cohort, the incidence of end-stage renal disease (ESRD) is increasing at the fastest rate, while the overall prevalence of CKD has grown from 10.3 to 13.1%. Notably, for individuals over 70 years, the increase in prevalence is especially marked, climbing from 37 to 47% (28). These findings further emphasize that with advancing age, the interaction between cardiovascular, renal, and metabolic diseases significantly elevates the risk of CKM syndrome.

Furthermore, our subgroup analysis elaborates on the CKM risk in specific high-risk populations. The risk of CKM varies significantly across individuals with different BMI and marital statuses. Obesity is strongly associated with atherosclerosis, coronary artery disease, and hypertension, with the risk of CVD increasing as BMI rises (29). Obesity also places additional burden on the kidneys, leading to hypertension, hyperglycemia, and hyperlipidemia, which in turn impair renal function (30). High BMI is typically linked to MetS (e.g., hyperglycemia, hyperlipidemia, abdominal obesity), which collectively exacerbates the risk of CVD and kidney disorders (31).

Marriage status is another important factor influencing cardiovascular, renal, and metabolic health through various social, psychological, and physiological mechanisms. Married or cohabiting individuals tend to have stronger social support systems and better health behaviors, resulting in a lower CKM risk. In contrast, unmarried or divorced individuals, due to lack of social support and higher life stress, are at a higher CKM risk. A review suggests that being married is linked to a reduced likelihood of experiencing factors that contribute to CVD and leads to improved general health (32). Consequently, intervention strategies must be customized to fit the attributes of various BMI and marital status categories to efficiently decrease the occurrence of CKM syndrome and elevate the patients’ quality of life.

The NHANES serves as a crucial instrument for assessing the health condition of the U.S. population and offers essential information for research in public health. Nevertheless, it is crucial to recognize certain limitations within this dataset. Initially, NHANES mainly functions as a cross-sectional survey, offering data based on a specific moment. This framework constrains its capability to determine causal links or monitor evolving changes over time. Additionally, some of the data in NHANES (e.g., lifestyle factors, dietary habits, smoking, and alcohol consumption) rely on self-reports, which may introduce biases or inaccuracies, particularly when respondents are asked about sensitive behaviors or health conditions. Furthermore, missing data, especially in long-term follow-ups, can compromise the reliability and accuracy of the analysis. To validate the results of this research and investigate the time-related dynamics of the connection between NHHR and CKM syndrome, upcoming studies ought to include longitudinal approaches or the integration of follow-up information. Moreover, integrating biological samples and objective measurements (such as urine or blood samples) could help verify the findings and reduce reliance on self-reported data. Additionally, methods such as multiple imputation or other techniques for handling missing data (e.g., maximum likelihood estimation) should be employed to mitigate the impact of missing data and enhance the robustness of the analysis. These approaches would improve our understanding of the factors associated with CKM syndrome and provide more accurate insights for future prevention and intervention strategies. Although the NHANES data constitutes a substantial database, its sample is limited to the American population. Consequently, the variations in genetic, cultural, and dietary factors among other populations remain unaddressed, which restricts the global applicability of the research findings.

## Conclusion

This research underscores the promise of NHHR as an uncomplicated and economical biomarker for evaluating the risk associated with CKM syndrome. Given the rising global prevalence of CKM syndrome, early identification of individuals at high risk using NHHR could help inform targeted prevention and intervention strategies, potentially reducing the burden of this multifactorial syndrome. Future research should focus on further validating NHHR as a predictive marker for CKM syndrome in diverse populations and exploring its utility in clinical practice. Additionally, the interplay between NHHR and other biomarkers of metabolic dysfunction warrants further investigation to better understand the complex pathophysiology of CKM syndrome.

## Data Availability

The original contributions presented in the study are included in the article/Supplementary material, further inquiries can be directed to the corresponding author.

## Footnotes

^1^ https://www.cdc.gov/nchs/nhanes
